# Association between pan-immune-inflammation value and Serum Klotho levels: a cross-sectional analysis of renal function mediation

**DOI:** 10.3389/fimmu.2025.1567367

**Published:** 2025-05-30

**Authors:** Mingjie Liu, Xuan Zhou, Han Xu, Yan Wang, Yuting Duan, Xiangru Guo, Bai Wei

**Affiliations:** ^1^ Department of Oncology, Liyuan Hospital, Tongji Medical College, Huazhong University of Science and Technology, Wuhan, Hubei, China; ^2^ Department of Nephrology, Tongji Hospital, Tongji Medical College, Huazhong University of Science and Technology, Wuhan, Hubei, China

**Keywords:** pan-immune-inflammation value, Klotho, NHANES, inflammation, renal function

## Abstract

**Introduction:**

Klotho has emerged as a promising biomarker of aging processes. Although inflammation is known to modulate Klotho expression, the relationship between Pan-Immune-Inflammation Value (PIV) and serum Klotho levels has not been investigated. This study aimed to examine the association between PIV and serum Klotho levels while exploring the potential mediating effects of renal function parameters.

**Methods:**

A cross-sectional analysis was conducted using data from the National Health and Nutrition Examination Survey (NHANES) 2007-2016. A total of 13,406 participants were included. Multiple linear regression models were used to evaluate the association between PIV and Klotho levels. Boruta feature selection method was used to identify variables included in the multivariable models. Smooth curve fitting was performed to examine potential non-linear relationships and threshold effect analysis was conducted using a two-piecewise linear regression model. Mediation analysis was conducted to assess the role of renal function indicators. Stratified analyses were performed to identify potential effect modifiers.

**Results:**

After fully adjusting for potential confounders, PIV showed a significant inverse association with serum Klotho levels (β=-0.11, 95%CI: -0.15, -0.07, *P <*0.0001). This relationship exhibited a U-shaped pattern with a threshold effect at PIV=800 (*P* for log-likelihood ratio = 0.003). Renal function indicators significantly mediated this association, with serum uric acid showing the strongest mediating effect (8.32%, *P <*0.0001), followed by estimated glomerular filtration rate (6.91%, *P <*0.0001), blood urea nitrogen (6.64%, *P <*0.0001), and serum creatinine (5.49%, *P <*0.0001). Cancer survivors exhibited significantly lower Klotho levels than non-cancer participants (813.74 ± 8.55 vs. 849.69 ± 5.08 pg/mL; *P* < 0.0001), as well as higher PIV values (320.07 ± 6.58 vs. 299.07 ± 3.44; *P* = 0.0059).

**Conclusion:**

Elevated PIV was found to be significantly associated with lower klotho levels, with renal function indicators playing a mediating role.

## Introduction

The Klotho protein, which is primarily secreted by the kidneys, has been demonstrated to possess many physiological functions, including the regulation of mineral metabolism, the inhibition of oxidative stress, and the delaying of cellular senescence (1). Previous studies have demonstrated that there is a progressive decline in Klotho expression levels with age (2). This decline is closely associated with the onset and progression of some age-related diseases, including cardiovascular disease, renal dysfunction, and neurodegenerative disease (3). The results of animal studies indicate that Klotho deficiency accelerates the emergence of aging-like phenotypes, whereas exogenous Klotho supplementation ameliorates various pathological conditions, which underscores its critical role in the regulation of aging (4). C-reactive protein (CRP) is a sensitive marker for detecting inflammation, and the literature indicates that it serves as a valuable indicator of the inflammatory levels associated with cardiovascular disease (5, 6). In a case-control study conducted by Martín-Núñez et al., the results demonstrated that reduced levels of soluble Klotho were linked to a pro-inflammatory state characterized by elevated CRP levels in patients with cardiovascular disease (7). Moreover, patients with chronic kidney disease (CKD) exhibit significantly reduced Klotho levels accompanied by elevated inflammatory cytokines, including interleukin-6 (IL-6) and tumor necrosis factor-alpha (TNF-α) (8).

Despite the established importance of Klotho in inflammatory pathways and aging, the precise interplay between Klotho levels and comprehensive inflammatory assessments, such as the Pan-Immune-Inflammation Value (PIV), has yet to be systematically explored. The PIV, derived from the product of the neutrophil count, platelet count, and monocyte count divided by the lymphocyte count, emerges as a novel inflammatory indicator that enables a comprehensive assessment of systemic inflammatory status (9). PIV is distinguished from traditional inflammatory markers (such as neutrophil-lymphocyte ratio (NLR) and platelet-lymphocyte ratio (PLR)), through its integration of multiple immune cell components, rather than focusing on limited cell types, thereby offering a more comprehensive assessment of systemic inflammatory and immune status (10). Research has shown that PIV exhibits superior predictive capability compared to conventional inflammatory markers across various clinical conditions, including cancer prognosis and cardiovascular diseases (11, 12). The prognostic value of PIV has been further validated in diverse pathological conditions, including nephropathy and age-related conditions (13, 14).

The kidney is one of the primary organs expressing membrane-bound Klotho protein and thus plays a pivotal role in modulating inflammatory responses and the aging process (15). The kidney-Klotho axis operates through multiple mechanisms, including regulation of oxidative stress, systemic inflammatory responses, fibrosis, and mineral homeostasis. Specifically, Klotho protects kidney function by partially inhibiting nuclear factor kappa B (NF-κB) activation to reduce inflammation, suppressing transforming growth factor-beta 1 (TGF-β1) signaling to attenuate renal fibrosis, and acting as a co-receptor for fibroblast growth factor 23 in calcium-phosphate metabolism (16–18). Collectively, these mechanisms contribute to maintaining renal function and delaying disease progression. The available body of evidence suggests a potential mediating role of renal function in the interplay between inflammation and Klotho regulation, given the frequent coexistence of decreased Klotho expression and heightened inflammatory status with impaired renal function (19). Nevertheless, the precise clinical mechanisms underlying this mediation remain to be elucidated. Additionally, cancer status significantly affects inflammatory responses and aging processes. Studies have shown that cancer patients commonly exhibit various physiological and metabolic changes, such as increased inflammation, oxidative stress, and reduced renal function, all of which may influence Klotho expression (20). However, few studies have systematically examined how cancer status affects the relationship between inflammation and Klotho level.

The existing literature indicated negative correlations between Klotho levels and various inflammatory markers, such as the CRP, Systemic Immune-Inflammation Index (SII), PLR, and NLR (21–23). However, the relationship between the PIV and Klotho levels remains unexplored. Understanding this relationship is significant, as it may reveal the potential role of inflammation in Klotho signaling and its link to related pathophysiological processes. Therefore, we hypothesized that there is a negative relationship between PIV and Klotho levels, suggesting that higher PIV may be associated with lower Klotho levels. To evaluate this hypothesis, our study analyzed data from the National Health and Nutrition Examination Survey (NHANES) conducted from 2007 to 2016. Our objective was to examine the association between PIV and serum Klotho levels and to assess how renal function indicators might moderate this relationship. Additionally, the study aimed to investigate whether the observed association varies among individuals with different cancer statuses. The findings have the potential to advance the theoretical understanding of the mechanisms connecting inflammation, kidney function, and the aging process, thereby offering new insights into the prevention and treatment of age-related diseases.

## Methods

### Study design and participants

NHANES is a nationally representative survey conducted by the National Center for Health Statistics (NCHS), employing a complex, multistage probability sampling design to assess the health and nutritional status of the non-institutionalized civilian US population (24). Ethical approval for the study was granted by the Institutional Review Board of the National Center for Health Statistics (NCHS), and all procedures were performed following the Declaration of Helsinki. All participants provided informed consent prior to their involvement in the study. Given that the NHANES dataset is publicly accessible and unmodified, this allowed for the waiver of additional informed consent requirements and ethical review processes. Standardized protocols were followed for all examinations and blood sample collections at Mobile Examination Centers (25). Detailed methodological information is publicly available on the NHANES website (https://www.cdc.gov/nchs/nhanes/).

**Figure 1:**
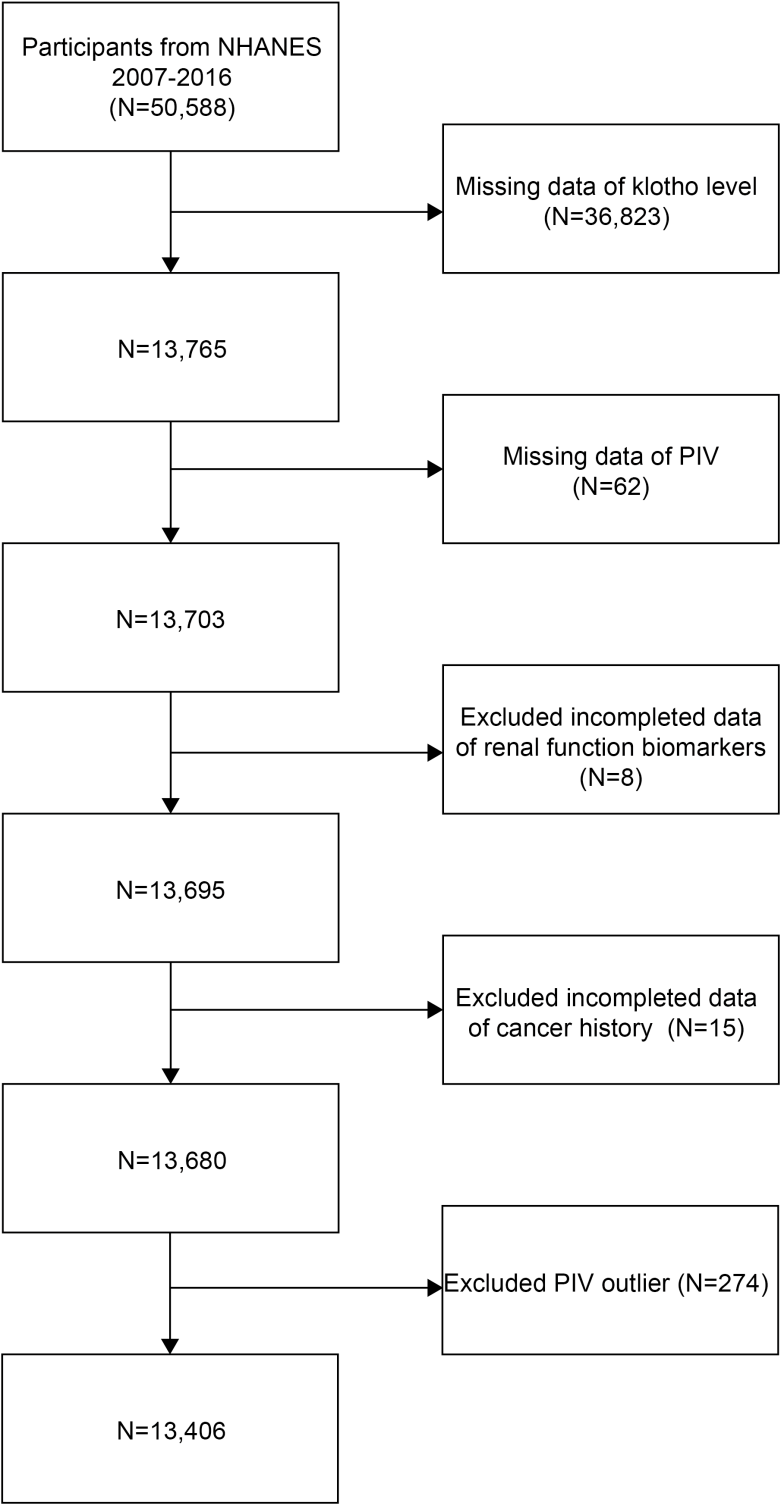
Flowchart of the sample selection.

This cross-sectional study analyzed data from the NHANES 2007-2016. Initially, 50,588 participants were recruited. Then, individuals with missing data on serum Klotho (n=36,823), PIV measurements (n=62), renal function biomarkers (n=8), and cancer history (n=15) were excluded from the study. Furthermore, to reduce the potential influence of extreme values and ensure robust statistical analysis, participants with PIV values outside the 1st and 99th percentiles (PIV <46.565 or >1226.782, n=274) were excluded. The final analytical sample consisted of 13,406 participants (**Figure 1**).

### Definition of PIV

The PIV was calculated using the following formula: PIV = neutrophil count × platelet count × monocyte count/lymphocyte count (all cell counts in ×10^9^/L) (26).

### Measurement of the Serum Klotho levels

Serum Klotho levels were measured using frozen samples collected from participants aged 40 to 79 years during the NHANES 2007–2016 examination. Samples were stored at -80°C until analysis. The serum Klotho levels were measured using a commercially available enzyme-linked immunosorbent assay (ELISA) kit (IBL International, Japan), following the protocol stipulated by the manufacturer (27). All samples were analyzed in duplicate, and the mean values were used for subsequent analyses.

### Definition of renal function indicators

The renal function of the subjects in this study was assessed using four key indicators: serum uric acid (UA), blood urea nitrogen (BUN), serum creatinine (Cr), and estimated glomerular filtration rate (eGFR). The eGFR was calculated according to the 2009 Chronic Kidney Disease Epidemiology Collaboration (CKD-EPI) equation (28). The equation is expressed as: eGFR = 141 × min(Scr/κ,1)α × max(Scr/κ,1)−1.209 × 0.993age × 1.018 (if female) × 1.159 (if black), where Scr represents serum creatinine in mg/dL; κ equals 0.7 for females and 0.9 for males; α equals −0.329 for females and −0.411 for males; min and max denote the minimum and maximum values of Scr/κ or 1, respectively.

### Assessment of covariates

Based on previous studies and clinical practice, this study took into account potential confounding factors that might influence the association between PIV and Klotho levels (29). The covariates encompassed demographic characteristics (age, sex, race/ethnicity), socioeconomic factors (family income to poverty ratio (PIR), education level, marital status), lifestyle behaviors (smoking status, drinking status), anthropometric measurements (body mass index (BMI)), and medical conditions (hypertension, diabetes, high cholesterol, and cancer history).

Information on demographics, lifestyle behaviors, and health conditions was collected through standardized NHANES questionnaires. Race was classified into five categories: Mexican American, Other Hispanic, Non-Hispanic White, Non-Hispanic Black, and Other Race. Education level was categorized as less than high school, high school graduate/General Education Development (GED), or more than high school. Marital status was defined as married/living with partner, widowed/divorced/separated, or never married. The smoking status was categorized as current, former, or never smokers based on lifetime smoking history and current smoking behavior (30). Current smokers are defined as individuals who have smoked ≥ 100 cigarettes and currently smoke (either daily or occasionally). Former smokers are those who have smoked ≥ 100 cigarettes in the past but no longer smoke, while never smokers are individuals who have smoked fewer than 100 cigarettes in their lifetime. Alcohol consumption was defined as the consumption of alcoholic beverages at least 12 times in the past year (yes/no). BMI was calculated as weight in kilograms divided by height in meters squared (kg/m²). The presence of medical conditions, including hypertension, diabetes, high cholesterol, and cancer, was ascertained through self-reported physician diagnoses.

### Statistical analyses

Statistical analyses accounted for the complex survey design of NHANES, incorporating appropriate sample weights (WTMEC2YR/5 for the 10-year sample) to ensure nationally representative estimates. According to the central limit theorem, when the sample size is sufficiently large, the distribution of sample means approaches a normal distribution (31). This property allows for the reasonable assumption of normality in the sample means during multivariable linear regression analyses, even when the original data do not fully adhere to a normal distribution. In this study, our sample size exceeds 10,000, which meets the requirements of the central limit theorem. Variance Inflation Factor (VIF) was used to assess multicollinearity, and all variables had VIF values less than 5, indicating that there is no significant multicollinearity issue (**Supplementary Table 1**). Continuous variables were expressed as weighted means ± standard errors (SE), and categorical variables as weighted percentages. Between-group comparisons were performed using survey-weighted linear regression for continuous variables and survey-weighted Chi-square tests for categorical variables. The study population was stratified into quartiles (Q1-Q4) based on PIV distribution. To examine the association between PIV and Klotho levels, weighted multiple linear regression analyses were conducted with PIV as both continuous and categorical variables. Results were expressed as Beta coefficients (β) with 95% confidence intervals (CI). Three sequential models were constructed: Model 1 (unadjusted); Model 2 (adjusted for age, sex, and race); and Model 3 (further adjusted for PIR, BMI, education level, marital status, smoking status, drinking status, hypertension, diabetes, high cholesterol, and cancer history). In this study, we utilized the Boruta feature selection method to identify all variables included in the multivariable models. The Boruta algorithm is a supervised feature selection technique based on random forests that minimizes the error of the random forest model, ultimately resulting in a subset of the most optimal features (32). Effect modification was assessed through stratified analyses by demographic and clinical characteristics, including sex, age (≤65, >65 years), race, PIR (<1.3, 1.3-3.5, ≥3.5), BMI categories (<25, 25-29.9, ≥30 kg/m²), smoking status (current, former, never), drinking status, and comorbidities. Potential non-linear relationships were evaluated using generalized additive models with smooth curve fitting. When non-linearity was detected, threshold effects were investigated using two-piecewise linear regression models, with optimal inflection points determined by likelihood ratio tests.

To evaluate whether renal function indicators (UA, BUN, Cr, and eGFR) mediated the relationship between PIV and Klotho, we utilized the R “mediation” package to assess direct, indirect, and total effects, while controlling for the covariates included in Model 3. Mediation was confirmed by the presence of significant indirect effects, total effects, and positive mediation proportions (33, 34). The calculation of the mediation proportion involved dividing the indirect effect by the sum of the indirect and direct effects, followed by multiplying by 100%. To enhance the robustness of our findings, we employed nonparametric bootstrap methods, specifically performing 1,000 resamples. The CIs were derived using the percentile method, which allows for the estimation of the variability in the indirect effects. This approach helps to address potential non-normality in the sampling distribution of the estimates and provides more reliable confidence intervals for the mediation effects. Furthermore, given the potential for cancer to modify inflammatory pathways, the study undertook additional analyses stratified by cancer status to examine potential effect modification in the PIV-Klotho association. All analyses were performed using EmpowerStats (version 4.2) and R software (version 4.2.0). Statistical significance was defined as *P* < 0.05 (two-sided). In our statistical analysis, we calculated the P-values for each primary outcome and concurrently reported 95% CI to provide a more comprehensive assessment of the reliability of the effects.

## Results

### Baseline characteristics of study participants according to PIV quartiles

This weighted analysis included 108,848,700 participants, who were classified into four quartiles based on their PIV values. The Klotho levels exhibited a gradual decline across these quartiles (**Table 1**). Additionally, higher PIV quartiles were associated with elevated creatinine levels and decreased eGFR, indicating a potential decline in renal function. Compared to participants in the lowest PIV quartile (Q1), those in the highest quartile (Q4) were more likely to be male, non-Hispanic white, older, have a higher BMI, possess an education level above high school, be married or living with a partner, consume alcohol, smoke currently, and have a lower PIR. As PIV levels increased, the prevalence of comorbidities such as hypertension, diabetes, and a history of cancer also rose, while the incidence of high cholesterol did not show significant differences.

**Table 1:** Baseline characteristics of study participants according to PIV Quartiles.

Variables	Q1	Q2	Q3	Q4	*P*-value
PIV	114.71 ± 0.63	196.70 ± 0.62	296.27 ± 0.99	555.50 ± 3.68	<0.0001
Klotho (pg/mL)	891.84 ± 9.58	848.91 ± 6.34	836.31 ± 7.93	811.43 ± 6.24	<0.0001
Age (years)	55.88 ± 0.24	55.71 ± 0.27	56.18 ± 0.27	56.69 ± 0.26	0.0288
Gender (%)					0.0009
Male	44.34	48.24	46.7	50.98	
Female	55.66	51.76	53.3	49.02	
Race (%)					<0.0001
Mexican American	7.03	6.84	7.02	6.17	
Other Hispanic	5.3	4.95	4.75	4.04	
Non-Hispanic White	63.48	72.82	75.27	78.43	
Non-Hispanic Black	16.03	8.52	7.3	5.45	
Other Race	8.15	6.87	5.67	5.91	
Education Level (%)					<0.0001
Less than High School	6.46	6.23	6.4	5.67	
High School or GED	33.07	28.65	32.18	35.88	
More than High School	60.47	65.12	61.42	58.45	
Marital Status (%)					0.0015
Married/Living with Partner	71.34	72.44	70.9	67.36	
Widowed/Divorced/Separated	17.88	18.55	20	21.93	
Never married	10.78	9.01	9.1	10.71	
PIR	3.24 ± 0.06	3.36 ± 0.06	3.24 ± 0.06	3.19 ± 0.06	0.0209
BMI (kg/m²)	28.49 ± 0.17	29.19 ± 0.17	29.80 ± 0.16	30.44 ± 0.17	<0.0001
Smoking Status (%)					<0.0001
Current	15.01	14.43	17.59	24.94	
Former	27.65	30.11	31.51	29.36	
Never	57.34	55.47	50.9	45.7	
Alcohol Consumption (%)					0.0202
Yes	74.51	78.81	78.36	77.77	
No	25.49	21.19	21.64	22.23	
Hypertension (%)					<0.0001
Yes	36.84	38.55	42.96	46.18	
No	63.16	61.45	57.04	53.82	
Diabetes (%)					<0.0001
Yes	11.71	11.7	13.06	16.74	
No	84.94	86.28	84.42	80.4	
Borderline	3.35	2.02	2.52	2.85	
High Cholesterol (%)					0.07
Yes	44.87	47.35	49.82	48.09	
No	55.13	52.65	50.18	51.91	
Cancer (%)					0.0117
Yes	10.91	12.92	14.13	14.91	
No	89.09	87.08	85.87	85.09	
Cr (mg/dL)	0.88 ± 0.01	0.89 ± 0.01	0.89 ± 0.01	0.93 ± 0.01	0.0019
BUN (mg/dL)	13.89 ± 0.14	14.16 ± 0.13	14.14 ± 0.13	14.50 ± 0.13	0.0088
UA (mg/dL)	5.31 ± 0.04	5.46 ± 0.04	5.47 ± 0.03	5.63 ± 0.03	<0.0001
eGFR (mL/min/1.73m²)	88.27 ± 0.45	87.19 ± 0.45	87.04 ± 0.42	85.10 ± 0.49	<0.0001

PIV, pan-immune-inflammation value; PIR, Family Income to Poverty Ratio; BMI, body mass index; Cr, serum creatinine; BUN, blood urea nitrogen; UA, Serum uric acid; eGFR, estimated glomerular filtration rate.

### Feature selection

**Figure 2:**
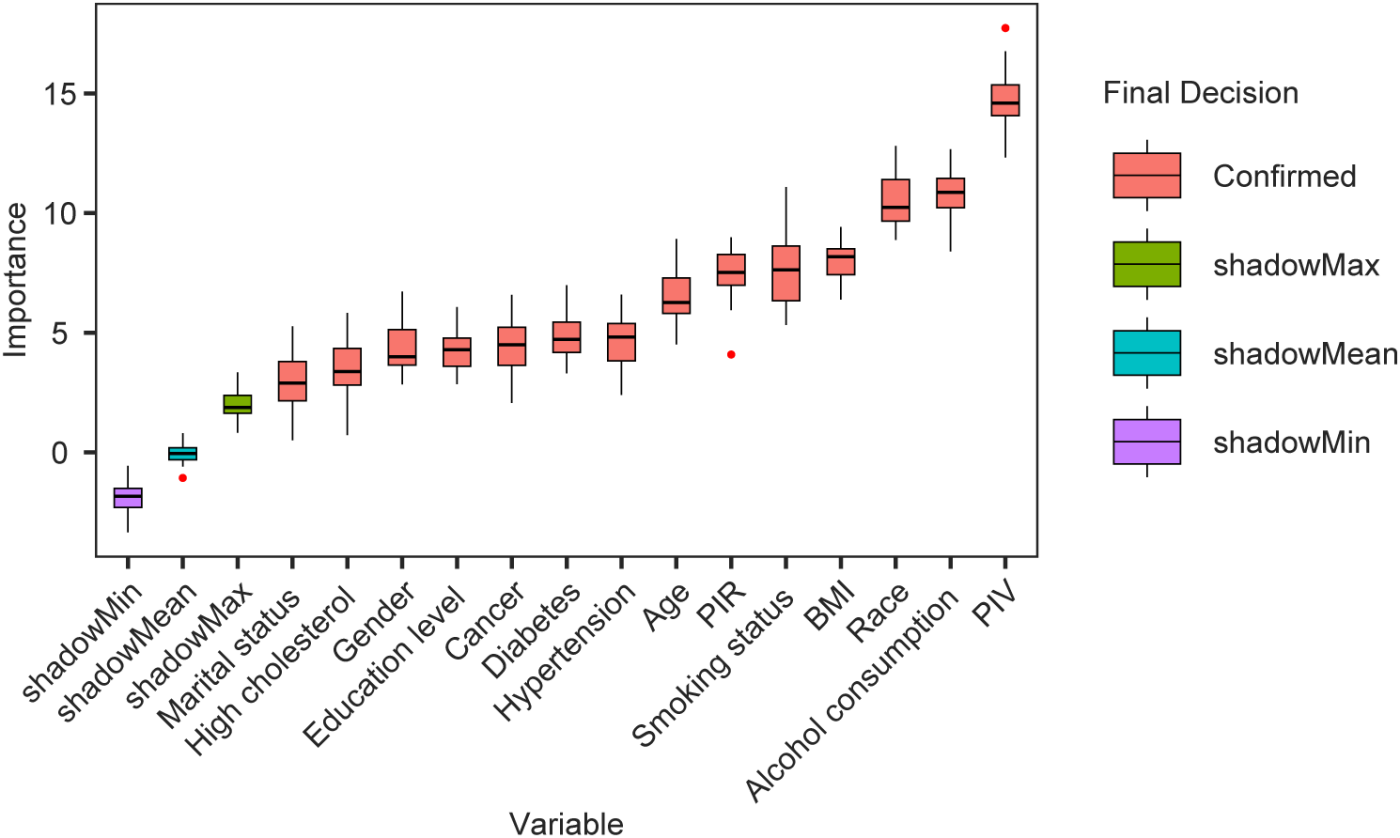
Predictor importance for klotho levels according to the Boruta algorithm.

Based on the results of feature selection using the Boruta algorithm, as shown in **Figure 2**, we identified 14 variables that were included in the multivariable models related to Klotho levels following 500 iterations. These variables, ranked by z-values, include PIV, drinking status, race, BMI, smoking status, PIR, age, hypertension, diabetes, cancer history, education level, gender, hyperlipidemia, and marital status. Consequently, we conclude that the most important predictor is PIV, followed by drinking behavior.

The horizontal axis displays the names of the variables, while the vertical axis represents their corresponding Z-values. The box plot illustrates the distribution of Z-values for each variable throughout the model calculations.

### Association between PIV and Klotho levels in total population

The association between PIV and Klotho levels was evaluated using three regression models (**Table 2**). In Model 1, each unit increase in PIV was associated with a significant decline in Klotho levels (β = -0.13, 95% CI: -0.16, -0.10, *P* < 0.0001). This inverse relationship remained significant in Model 2 (β = -0.11, 95% CI: -0.14, -0.08, *P* < 0.0001), and persisted in Model 3 (β = -0.11, 95% CI: -0.15, -0.07, *P* < 0.0001).

**Table 2:** Association between PIV Quartiles and Klotho levels in total population.

PIV	β (95% CI) *P*-value		
	Model 1	Model 2	Model 3
Per 1 increment	-0.13 (-0.16, -0.10) <0.0001	-0.11 (-0.14, -0.08) <0.0001	-0.11 (-0.15, -0.07) <0.0001
Q1	**Reference**	**Reference**	**Reference**
Q2	-42.93 (-62.46, -23.40) 0.0001	-37.54 (-57.69, -17.38) 0.0005	-37.62 (-60.67, -14.57) 0.0022
Q3	-55.53 (-76.51, -34.54) <0.0001	-48.75 (-70.50, -27.01) <0.0001	-46.37 (-69.02, -23.72) 0.0002
Q4	-80.41 (-100.16, -60.65) <0.0001	-69.68 (-90.16, -49.19) <0.0001	-67.26 (-91.35, -43.18) <0.0001
*P* for trend	<0.0001	<0.0001	<0.0001

Model 1, Unadjusted.

Model 2, Adjusted for age, sex, and race.

Model 3, Adjusted for age, sex, race, PIR, BMI, education level, marital status, smoking status, drinking status, hypertension, diabetes, high cholesterol, and cancer history.

PIV, pan-immune-inflammation value; PIR, Family Income to Poverty Ratio; BMI, body mass index.

Further analysis revealed a dose-dependent relationship between PIV quartiles and Klotho levels. In Model 3, participants in Q4 exhibited the most significant decline in Klotho levels (β = -67.26, 95% CI: -91.35, -43.18, *P* < 0.0001). A significant trend across quartiles was observed in all models (all *P* for trend < 0.0001), indicating a robust and consistent inverse association between PIV and Klotho levels.

### U-shaped pattern and threshold effect of PIV on Klotho levels

**Figure 3:**
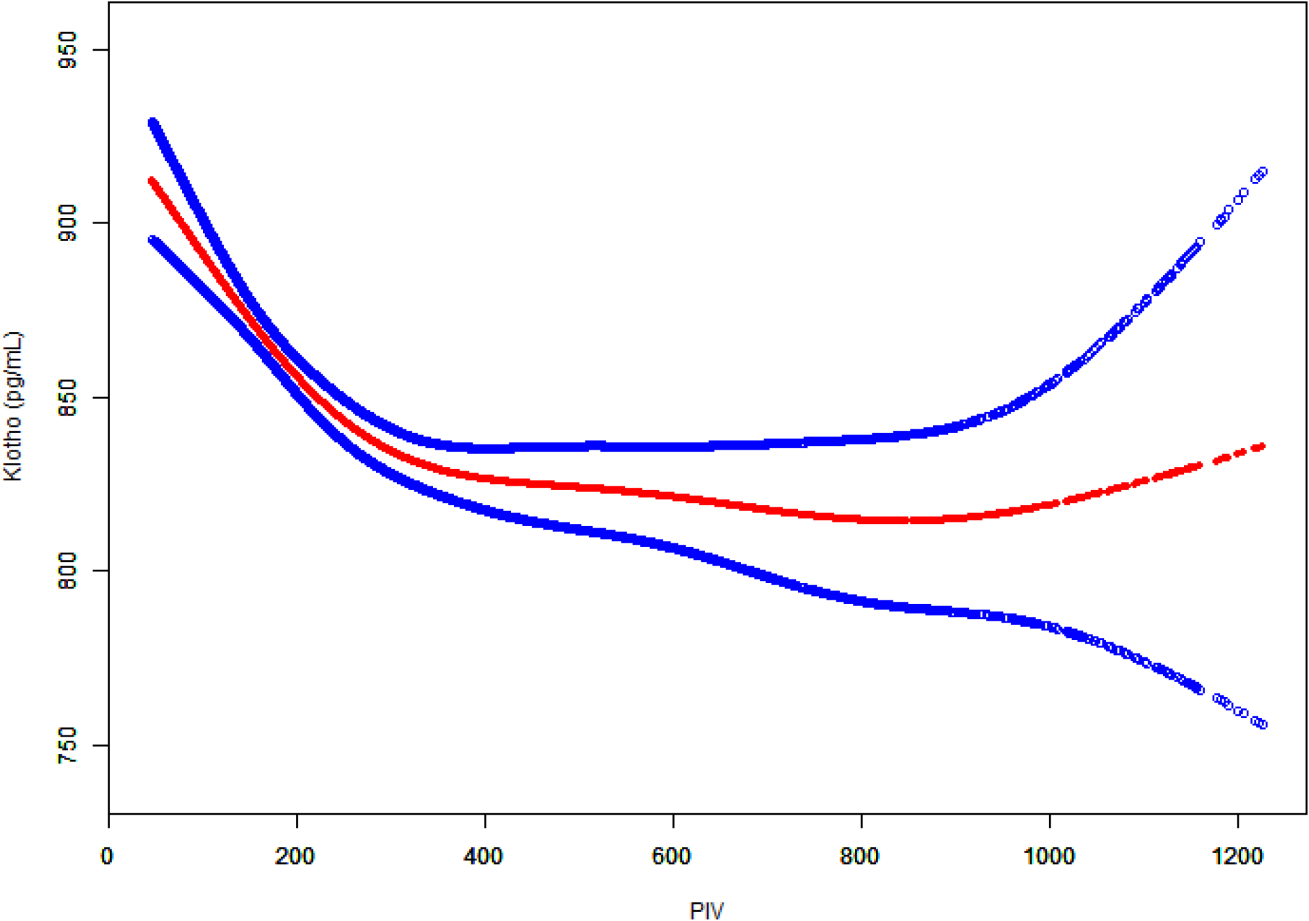
Relationship between Klotho levels and PIV in the total population.

To further investigate the potential non-linear relationship between PIV and Klotho levels, generalized additive models were employed for smooth curve fitting (**Figure 3**). The analysis yielded a U-shaped association, whereby Klotho levels exhibited a steady decline with rising PIV until approximately 800, followed by a gradual increase.

Two-piecewise linear regression analysis identified a threshold effect at PIV = 800 (**Table 3**). In the segment preceding this threshold, a negative association was observed between PIV and Klotho levels (β = -0.13, 95% CI: -0.17, -0.10, *P* < 0.0001). In the segment after the threshold, the association shifted in a positive direction but did not reach statistical significance (β = 0.19, 95% CI: -0.01, 0.39, *P* = 0.0631). The likelihood ratio test demonstrated that the two-piecewise linear regression model provided a better fit compared to the single linear model (*P* = 0.003). These findings suggested that the association between PIV and Klotho levels was non-linear with an inflection point at PIV = 800.

**Table 3:** Threshold effect analysis of the association between PIV and Klotho levels.

Model	Adjusted β (95% CI), *P*-value
Fitting by the standard linear model	-0.11 (-0.14, -0.07) <0.0001
Fitting by the two-piecewise linear model	
Inflection point	800
<Inflection point	-0.13 (-0.17, -0.10) <0.0001
>Inflection point	0.19 (-0.01, 0.39) 0.0631
*P* for Log-likelihood ratio	0.003

Adjusted for potential confounders as specified in Model 3.

The graph depicts PIV values on the x-axis and Klotho levels (pg/ml) on the y-axis. The red dots represent the fitted curve and the blue dots indicate the 95% CI. Adjusted for potential confounders as specified in Model 3.

### Subgroup analyses

**Figure 4:**
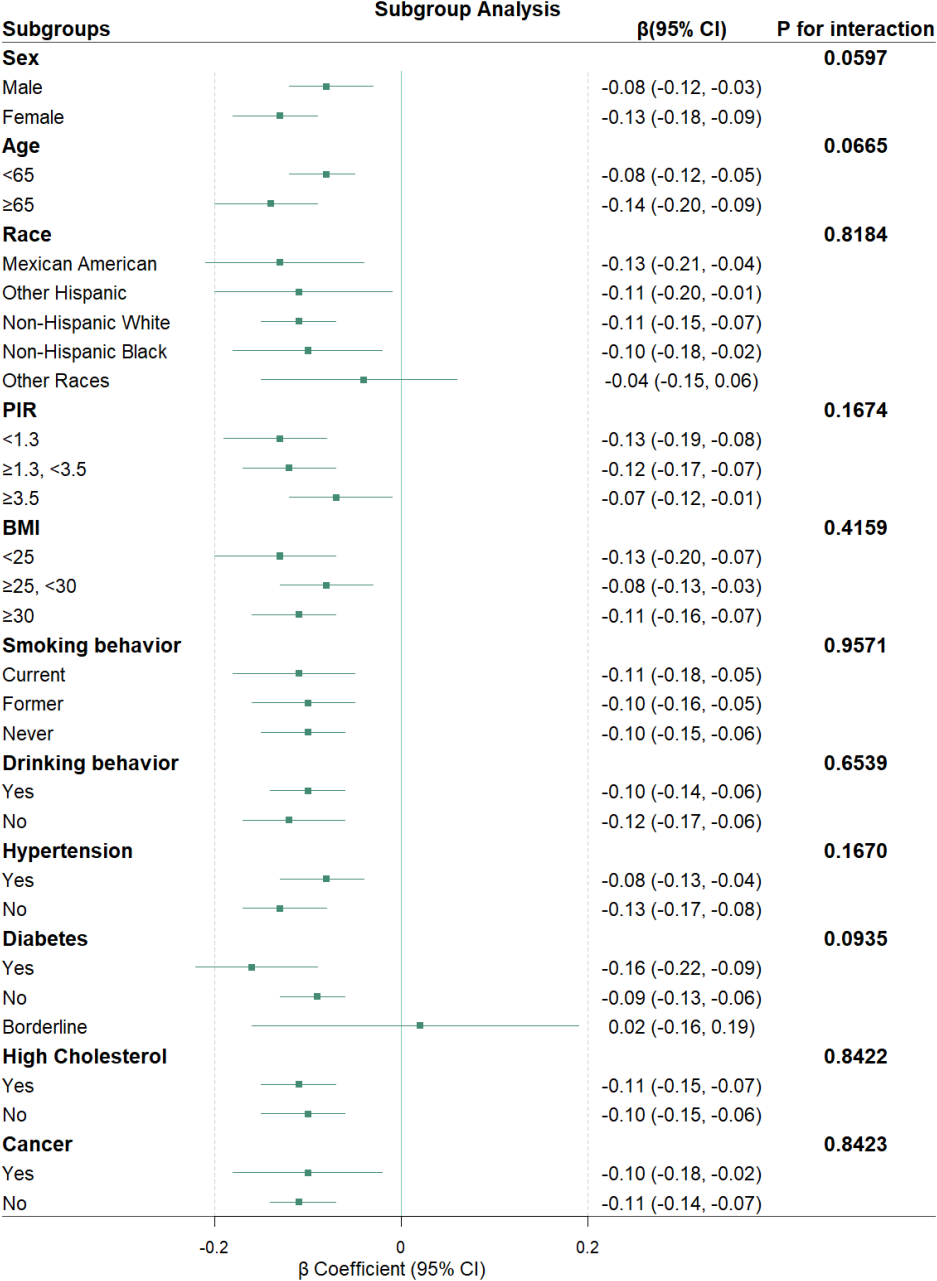
Stratified analyses of associations between PIV and klotho levels.

Subgroup analyses were conducted to evaluate the consistency of the association between PIV and Klotho levels across various population characteristics (**Figure 4**). The results indicate that, in most subgroups, there is a consistent relationship between PIV and Klotho levels, revealing a statistically significant negative correlation. Furthermore, the association between PIV and Klotho levels remained consistent across all subgroups, with all *P*-values for interaction exceeding 0.05.

Forest plots showing β coefficients (green squares) and 95% CI (horizontal lines). All models were adjusted for potential confounders as specified in Model 3, except for the stratification factor in each subgroup analysis.

### Associations of renal function biomarkers with PIV and Klotho level

In Model 1, higher levels of Cr, BUN, and UA were positively associated with PIV but negatively associated with Klotho levels (all *P*<0.0001) (**Tables 4**, **5**). Conversely, eGFR demonstrated a negative association with PIV but a positive association with Klotho levels (both *P*<0.0001). These associations remained consistent in Model 2 (all *P*<0.05). In Model 3, Cr, UA, and eGFR maintained significant associations with both PIV and Klotho levels (all *P*<0.05), while BUN was no longer significantly associated with PIV (β = 0.78, 95% CI: -0.16, 1.72, *P* = 0.1077) but retained a significant inverse association with Klotho levels (β = -3.29, 95% CI: -4.66, 1.92, *P*<0.0001).

**Table 4:** The associations between PIV and renal function indicators.

PIV	β (95% CI) *P*-value		
	Model 1	Model 2	Model 3
Cr	23.34 (12.65, 34.04) <0.0001	22.74 (12.22, 33.27) <0.0001	20.26 (8.24, 32.28) 0.0016
BUN	1.59 (0.86, 2.32) <0.0001	0.92 (0.19, 1.65) 0.0161	0.78 (-0.16, 1.72) 0.1077
UA	11.08 (7.91, 14.24) <0.0001	11.01 (7.26, 14.76) <0.0001	5.34 (0.68, 10.01) 0.0287
eGFR	-0.68 (-0.91, -0.46) <0.0001	-0.52 (-0.80, -0.24) 0.0006	-0.51 (-0.84, -0.18) 0.0039

Model 1: Unadjusted.

Model 2: Adjusted for age, sex, and race.

Model 3: Adjusted for age, sex, race, PIR, BMI, education level, marital status, smoking status, drinking status, hypertension, diabetes, high cholesterol, and cancer history.

PIV, pan-immune-inflammation value; Cr, serum creatinine; BUN, blood urea nitrogen; UA, Serum uric acid; eGFR, estimated glomerular filtration rate.

**Table 5:** The associations between renal function indicators and klotho levels.

Klotho	β (95% CI) *P*-value		
	Model 1	Model 2	Model 3
Cr	-68.28 (-93.88 -42.69) <0.0001	-58.87 (-83.86 -33.88) <0.0001	-59.94 (-90.43, -29.46) 0.0003
BUN	-4.71 (-5.98 -3.44) <0.0001	-3.01 (-4.20 -1.81) <0.0001	-3.29 (-4.66, -1.92) <0.0001
UA	-30.67 (-36.82 -24.53) <0.0001	-29.34 (-35.75 -22.93) <0.0001	-30.02 (-36.64, -23.41) <0.0001
eGFR	2.16 (1.84 2.47) <0.0001	1.87 (1.51 2.24) <0.0001	1.89 (1.45, 2.32) <0.0001

Model 1: Unadjusted.

Model 2: Adjusted for age, sex, and race.

Model 3: Adjusted for age, sex, race, PIR, BMI, education level, marital status, smoking status, drinking status, hypertension, diabetes, high cholesterol, and cancer history.

Cr, serum creatinine; BUN, blood urea nitrogen; UA, Serum uric acid; eGFR, estimated glomerular filtration rate.

### The mediating role of renal function

**Figure 5:**
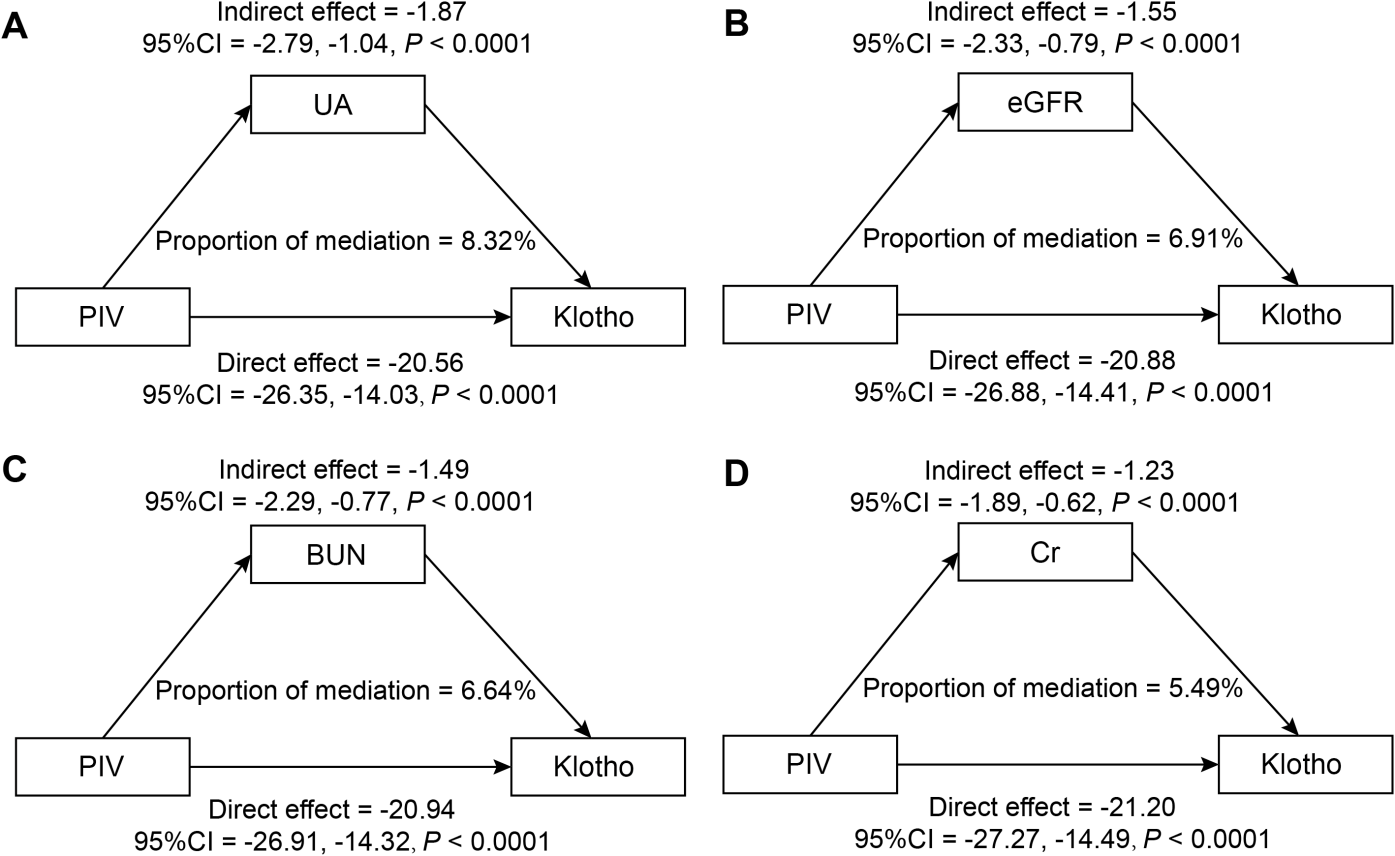
Mediation analysis of renal function indicators in the association between PIV and Klotho levels (pg/ml): **(A)** UA (mg/dL); **(B)** eGFR (mL/min/1.73m²); **(C)** BUN (mg/dL); **(D)** Cr (mg/dL).

The mediation analysis, after adjusting for all potential confounding factors in Model 3, revealed significant mediating effects of renal function indicators on the relationship between PIV and Klotho levels (**Figure 5**). Among the four renal function indicators examined, UA demonstrated the strongest mediating effect, accounting for 8.32% of the total effect (95% CI: 4.55%, 13.30%, *P* < 0.0001). This was followed by eGFR, mediating 6.91% of the total effect (95% CI: 3.43%,11.68%, *P* < 0.0001). BUN and Cr showed relatively smaller but still significant mediating effects, with mediation proportions of 6.64% (95% CI: 3.33%, 11.06%, *P* < 0.0001) and 5.49% (95% CI: 2.62%, 9.31%, *P* < 0.0001), respectively. The results of the mediation analysis are presented in **Supplementary Table 2**.

Adjusted for potential confounders as specified in Model 3.

### Cancer-stratified sensitivity analyses

A subsequent sensitivity analysis stratified by cancer status revealed significant differences between cancer survivors and non-cancer participants (**Supplementary Table 3**). Cancer survivors exhibited significantly lower klotho levels (813.74 ± 8.55 vs. 849.69 ± 5.08 pg/mL, *P* < 0.0001) and higher PIV values (320.07 ± 6.58 vs. 299.12 ± 3.44, *P* = 0.0059). They were also older and presented with a higher burden of cardiovascular risk factors, including a higher prevalence of hypertension, diabetes, and hypercholesterolemia (all *P*<0.05).

The inverse association between PIV and klotho levels remained significant in both populations, though with varying magnitudes (**Supplementary Table 4**). Among cancer survivors, Klotho levels in Q4 showed the most significant decline compared to those in Q1, with a coefficient of β = -59.13 (95% CI: -116.43, -1.83, *P* = 0.0478; *P* for trend = 0.0233). Similarly, non-cancer participants exhibited a progressive decrease in Klotho levels across PIV quartiles, with the most substantial reduction observed in Q4 (β = -67.82, 95% CI: -94.34, -41.29, *P* < 0.0001; *P* for trend < 0.0001).

**Figure 6:**
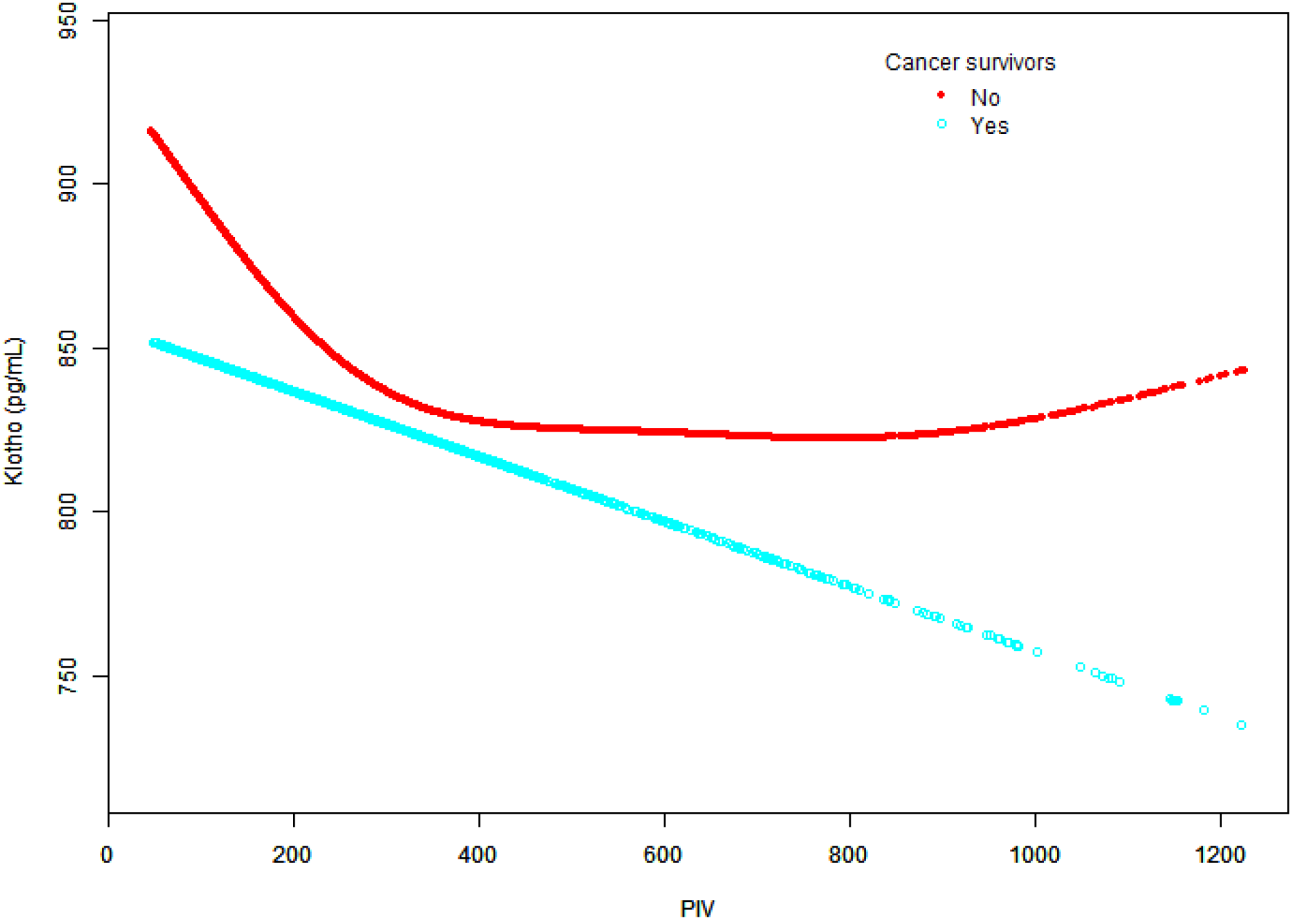
Relationship between Klotho levels and PIV in cancer survivors and non-cancer participants.

Smooth curve fitting revealed distinct patterns between groups (**Figure 6**). Non-cancer participants showed an initial decline in klotho levels that plateaued at higher PIV values, while cancer survivors demonstrated a more pronounced and continuous decline, particularly at higher PIV values.

The graph depicts PIV values on the x-axis and Klotho levels (pg/ml) on the y-axis. Two distinct trend lines are presented: cancer survivors (blue dots) and non-cancer participants (red dots). Adjusted for potential confounders as specified in Model 3, except for cancer status.

## Discussion

This study analyzed the relationship between PIV and Klotho levels for the first time. In this large-scale cross-sectional study of 13,406 US adults (weighted N=108,848,700), we found a robust inverse association between PIV and serum Klotho levels that persisted after comprehensive adjustment for potential confounders. Participants in Q1 exhibited significantly lower Klotho levels compared to those in Q4 (811.43 ± 6.24 vs. 891.84 ± 9.58 pg/mL, *P*<0.0001). Notably, this relationship demonstrated a U-shaped pattern with a threshold effect at PIV=800 (*P* for log-likelihood ratio = 0.003), suggesting a complex, non-linear interaction between systemic inflammation and Klotho expression. The association was consistent across different subgroups. Furthermore, our mediation analysis revealed that renal function indicators, particularly UA and eGFR, significantly mediated this relationship, accounting for up to 8.32% and 6.91% of the total effect, respectively (both *P* < 0.0001). These findings extend the current understanding of the intricate relationship between inflammation and aging-related proteins while highlighting the crucial role of renal function in this interaction.

The potential molecular mechanisms underlying the relationship between PIV and Klotho are multifaceted. As a comprehensive marker of systemic inflammation, PIV integrates immune cell counts (neutrophils, monocytes, platelets, and lymphocytes), offering advantages over single inflammatory markers in reflecting systemic inflammatory status (35). Previous studies have demonstrated that pro-inflammatory cytokines, particularly TNF-α and Interferon-gamma (IFN-γ), downregulate Klotho expression through the NF-κB signaling pathway (36–38). Under inflammatory conditions, cytokines such as TNF-α, Interleukin-1 beta (IL-1β), and IL-6 modulate Klotho levels through interconnected mechanisms, including direct suppression of Klotho gene transcription, activation of NF-κB signaling pathways, and disruption of cellular anti-inflammatory networks (39, 40).

Conversely, Klotho itself has potent anti-inflammatory properties through several mechanisms. It has been demonstrated that Klotho suppresses the NF-κB signaling pathway, thereby reducing the production of pro-inflammatory cytokines such as IL-6 and TNF-α (41, 42). Moreover, Klotho has been shown to exhibit a critical regulatory mechanism involving Toll-like receptor 4 (TLR4), a key pattern recognition receptor in inflammatory responses (43). Studies have shown that Klotho can suppress TLR4-mediated inflammatory signaling through its deglycosylation activity, effectively reducing TLR4 protein accumulation and subsequent pro-inflammatory cascade activation (44). By attenuating TLR4 signaling, Klotho effectively diminishes the recruitment of inflammatory cells and prevents excessive inflammatory responses. Furthermore, Klotho promotes M2 macrophage polarization, thereby enhancing anti-inflammatory responses (45, 46). Its regulatory role extends to the TGF-β1 signaling pathway, where it prevents epithelial-mesenchymal transition and mitigates tissue fibrosis (47, 48). In addition, recent evidence also suggests that Klotho enhances cellular antioxidant capacity by activating the nuclear factor erythroid 2-related factor 2 (Nrf2) signaling pathway, thus protecting cells from oxidative stress-induced damage (49, 50).

In this study, we observed a significant negative association between the systemic inflammation marker PIV and serum Klotho levels, suggesting that Klotho levels decrease with chronic inflammation. This finding supports the hypothesis that Klotho may act as a potential anti-inflammatory factor. However, other studies indicate that an increase in inflammation is not always associated with a decrease in Klotho levels (51). For instance, Alvarez-Cienfuegos et al. reported that patients with rheumatoid arthritis (RA) exhibited significantly higher levels of Klotho compared to healthy controls (52). These findings suggest that the elevation of Klotho may be a compensatory response to systemic inflammation in RA patients, with its anti-inflammatory properties potentially playing an important role in inflammatory states. Additionally, their study found a positive correlation between Klotho levels and anti-citrullinated protein antibodies (ACPA) as well as rheumatoid factors, indicating that pathologically elevated Klotho may be related to disease activity. This discrepancy highlights the complexity of the relationship between Klotho and systemic inflammation, emphasizing the need for further research to elucidate the mechanisms of Klotho in different disease contexts. Longitudinal studies are also necessary to clarify the temporal dynamics between Klotho and inflammation markers. Moreover, due to the cross-sectional design of the present study, it has not been possible to establish a causal relationship between PIV and Klotho levels. However, should a causal relationship be demonstrated between inflammation and Klotho, this might suggest that increasing endogenous Klotho secretion or providing exogenous Klotho supplementation could have significant clinical implications for treating chronic inflammation (53). Therefore, further prospective studies are needed to validate this hypothesis.

Additionally, previous research has identified an L-shaped association between SII and Klotho levels, revealing an inflection point at 255 pg/ml beyond which the relationship plateaus (29). This finding suggested that the regulatory interaction between inflammation and Klotho expression may involve complex threshold-dependent mechanisms. Similarly, our analysis revealed a distinctive threshold effect at PIV = 800 in the overall population, where the previously significant negative association between PIV and Klotho levels became attenuated. This phenomenon may be attributed to several interconnected mechanisms. Firstly, the deglycosylation activity of Klotho, which is crucial for the degradation of TLR4, may be compromised at elevated inflammatory states, further reducing TLR4 protein turnover and subsequent amplification of inflammatory signaling pathways (44). As PIV levels rise above the threshold, the regulatory capacity of Klotho may reach a saturation point, diminishing its ability to counteract TLR4-mediated inflammation. Moreover, feedback mechanisms of TLR4 signaling potentially create a self-perpetuating inflammatory cycle. As PIV levels rise, the increased TLR4 activation may further suppress Klotho expression, weakening its protective cellular mechanisms (54–56).

Notably, cancer survivors exhibit a distinct pattern in the PIV-Klotho relationship compared to non-cancer participants. While non-cancer individuals display an initial decline in Klotho levels at higher PIV values followed by stabilization, cancer survivors demonstrate a more pronounced and sustained decrease in Klotho levels at elevated PIV values. The NF-κB signaling pathway likely plays a crucial role in this linear relationship in cancer survivors. Existing research indicates that pro-inflammatory cytokines can suppress Klotho gene expression by activating NF-κB, a mechanism particularly evident in high-inflammatory states associated with cancer (57, 58). Animal research evidence further supports a direct relationship between Klotho expression and cancer progression, suggesting that Klotho suppression correlates with higher histologic grades (*P* = 0.013) and increased Ki-67 expression (*P* = 0.024) (59). These findings suggest that Klotho suppression is driven by cancer-related inflammation, which generates a unique inflammatory environment through interactions between malignant cells and the tumor microenvironment (60, 61). While these observations provide valuable insights, they should be interpreted cautiously due to the cross-sectional study design, which inherently limits causal inference. Further longitudinal research is essential to elucidate the molecular mechanisms underlying these intricate interactions.

The mediating effects of renal function parameters provide critical insights into the complex interactions within the PIV-Klotho axis. Inflammatory factors can directly damage glomerular and tubular cells, leading to a progressive decline in kidney function (62). Meanwhile, deteriorating kidney function significantly reduces Klotho production, consequently exacerbating inflammatory responses and kidney damage (63). A previous study revealed a significant negative correlation between UA and α-Klotho levels, demonstrating its critical intermediary role in the testosterone-α-Klotho axis (64). These findings suggested that UA was involved in the interaction mechanism between metabolic regulation and Klotho expression. Consistent with these insights, the present study substantiated the pivotal role of renal function parameters in mediating inflammation levels and Klotho expression. Our results showed that UA exhibited the most pronounced mediating effect, accounting for 8.32% of the total effect, with eGFR following closely at 6.91%. Mechanistically, UA has a dual role: at low concentrations, it has antioxidant properties; however, at high concentrations, it may induce oxidative stress (65). Elevated UA induces oxidative stress in renal tubular epithelial cells, establishing a deleterious positive feedback loop that progressively compromises the antioxidative protective mechanisms through Klotho (66). Specifically, elevated UA levels activate the NF-κB signaling pathway, thereby promoting inflammatory responses and subsequently suppressing Klotho gene expression (67, 68). A comprehensive systematic review and meta-analysis found a significant positive correlation between soluble α-Klotho levels and eGFR among CKD patients (69). Similarly, Ferri et al. revealed a significant increasing trend in eGFR across Klotho gene mRNA tertiles in patients with CKD. The highest tertile demonstrated improved eGFR (42.3 mL/min/1.73 m²) compared to the lowest tertile (36.7 mL/min/1.73 m²) (70). These findings substantiated that reduced eGFR is closely associated with decreased Klotho expression. Collectively, metabolic dysregulation in renal dysfunction triggers a cascade of pathological changes, characterized by toxic metabolite accumulation and an inflammatory positive feedback loop (71, 72). These possible alterations may directly influence Klotho synthesis and cellular signaling pathways regulating its expression and protective functions.

In conclusion, the present study demonstrated that the relationship between PIV and serum Klotho levels exhibits a U-shaped pattern, with renal function parameters (particularly UA and eGFR) acting as significant mediators. It is also noteworthy that the PIV-Klotho association exhibited a threshold effect at PIV=800. Furthermore, cancer survivors exhibited a sustained linear decrease in Klotho levels with increasing PIV, while non-cancer survivors demonstrated a U-shaped relationship. The findings of this study underscore the significance of monitoring inflammatory status and renal function parameters in clinical settings. Furthermore, in patients with impaired renal function, maintaining Klotho levels may be particularly important given its protective effects on kidney function. Future research should elucidate the molecular mechanisms underlying the complex relationship between systemic inflammation and Klotho expression, focusing on the mediating effects of renal function and the different patterns observed in different pathological conditions.

### Strengths and limitations

The present study was distinguished by several notable strengths. Firstly, it was the first study to investigate the correlation between PIV and Klotho levels. The research identified a statistically significant U-shaped relationship between PIV and Klotho expression with a threshold effect. Furthermore, the study revealed the mediating effect of renal function indicators, specifically UA and eGFR. The mediation analysis provided valuable insights into the potential mechanistic pathways underlying the interplay between inflammatory processes, kidney function, and Klotho protein regulation, thereby suggesting potential therapeutic targets for further research.

However, several limitations of the study should be acknowledged. Firstly, as a cross-sectional study, it cannot establish causal relationships among the variables since data were collected at a single time point. Consequently, the directionality remains unclear; systemic inflammation, as indicated by PIV, may influence Klotho levels, or Klotho levels may influence systemic inflammation. Therefore, longitudinal studies are recommended to clarify the temporal dynamics of this association and strengthen causal inferences. Additionally, uncontrolled confounding variables, such as genetic factors, dietary habits, and medication use, limit the ability to control for these influences when studying the relationship between systemic inflammation and Klotho expression. Additionally, recall bias may impact results, especially concerning self-reported lifestyle factors or health conditions. Participants may struggle to accurately recall past events or behaviors, leading to misclassification and potential inaccuracies in association estimates. This bias complicates the interpretation of findings by introducing additional variability not accounted for in the analysis. Furthermore, the sample from the NHANES database primarily represents the U.S. population, raising concerns about selection bias and limiting the generalizability of the findings to other ethnic or socio-demographic groups. Given these limitations, caution is warranted in extrapolating the findings to broader populations, as biological responses to inflammation and variations in Klotho levels may differ significantly across diverse ethnic groups and geographical contexts. Notwithstanding these limitations, the study offers novel insights into the intricate interplay between systemic inflammation and Klotho expression. By unveiling the complex relationship between inflammation and Klotho, this study provided valuable insights for future anti-aging research and potential intervention strategies. Future studies should consider multi-center, larger sample-size longitudinal designs that comprehensively collect a broader range of variables to validate the generalizability of the conclusions.

## Conclusion

The findings revealed a U-shaped relationship between PIV and Klotho levels, partially mediated by renal function indicators. These results emphasized the importance of monitoring renal function markers when considering the impact of systemic inflammation on the aging process.

## Data Availability

Publicly available datasets were analyzed in this study. This data can be found here: The survey data are publicly available on the website: https://wwwn.cdc.gov/Nchs/Nhanes/.

## References

[1] Neyra JA Hu MC Moe OW. Klotho in clinical nephrology: diagnostic and therapeutic implications. Clin J Am Soc Nephrol. (2020) 16:162–76. doi: 10.2215/CJN.02840320 PMC7792642 32699047

[2] Li H Miao S Zhang M Zhang P Li Y-B Duan R-S. U-shaped association between serum klotho and accelerated aging among the middle-aged and elderly US population: A cross-sectional study. BMC Geriatrics. (2023) 23(1):780. doi: 10.1186/s12877-023-04479-9 38017397 PMC10685632

[3] Kanbay M Brinza C Ozbek L Guldan M Sisman U Copur S. The association between klotho and kidney and cardiovascular outcomes: a comprehensive systematic review and meta-analysis. Clin Kidney J. (2024) 17:sfae255. doi: 10.1093/ckj/sfae255 39281418 PMC11398896

[4] Takenaka T Hasan A Marumo T Kobori H Inoue T Miyazaki T. Klotho supplementation attenuates blood pressure and albuminuria in murine model of IgA nephropathy. J Hypertens. (2021) 39:1567–76. doi: 10.1097/HJH.0000000000002845 33758157

[5] Kaplangoray M Toprak K Aslan R Deveci E Gunes A Ardahanli İ. High CRP-albumin ratio is associated high thrombus burden in patients with newly diagnosed STEMI. Med (Baltimore). (2023) 102:e35363. doi: 10.1097/MD.0000000000035363 PMC10578711 37832116

[6] Ardahanlı İ. The C-reactive protein to albumin ratio may be an inflammatory indicator for the coronary slow flow phenomenon. Lokman Hekim Health Sci. (2022) 3(1):32–7. doi: 10.14744/lhhs.2022.80006

[7] Martín-Núñez E Donate-Correa J Ferri C López-Castillo Á Delgado-Molinos A Hernández-Carballo C. Association between serum levels of Klotho and inflammatory cytokines in cardiovascular disease: a case-control study. Aging. (2020) 12:1952–64. doi: 10.18632/aging.102734 PMC7053623 31986490

[8] Wang S Xu Q Zhang Y Jiang X Wang N Hu Y. The FGF23-Klotho axis promotes microinflammation in chronic kidney disease. Cytokine. (2024) 184:156781. doi: 10.1016/j.cyto.2024.156781 39454251

[9] Jin C Li X Luo Y Zhang C Zuo D. Associations between pan-immune-inflammation value and abdominal aortic calcification: a cross-sectional study. Front Immunol. (2024) 15:1370516. doi: 10.3389/fimmu.2024.1370516 38605946 PMC11007162

[10] Murat B Murat S Ozgeyik M Bilgin M. Comparison of pan-immune-inflammation value with other inflammation markers of long-term survival after ST-segment elevation myocardial infarction. Eur J Clin Invest. (2023) 53:e13872. doi: 10.1111/eci.13872 36097823

[11] Kaplangoray M Toprak K Deveci E Caglayan C Şahin E. Could pan-immune-inflammation value be a marker for the diagnosis of coronary slow flow phenomenon? Cardiovasc Toxicol. (2024) 24:519–26. doi: 10.1007/s12012-024-09855-4 PMC11076385 38622332

[12] Li F Wang Y Dou H Chen X Wang J Xiao M. Association of immune inflammatory biomarkers with pathological complete response and clinical prognosis in young breast cancer patients undergoing neoadjuvant chemotherapy. Front Oncol. (2024) 14:1349021. doi: 10.3389/fonc.2024.1349021 38380360 PMC10877026

[13] Okyar Baş A Güner M Ceylan S Hafızoğlu M Şahiner Z Doğu BB. Pan-immune inflammation value; a novel biomarker reflecting inflammation associated with frailty. Aging Clin Exp Res. (2023) 35:1641–9. doi: 10.1007/s40520-023-02457-0 37289361

[14] Kazan DE Kazan S. Systemic immune inflammation index and pan-immune inflammation value as prognostic markers in patients with idiopathic low and moderate risk membranous nephropathy. Eur Rev Med Pharmacol Sci. (2023) 27:642–8. doi: 10.26355/eurrev_202301_31065 36734708

[15] Tanriover C Copur S Mutlu A Peltek IB Galassi A Ciceri P. Early aging and premature vascular aging in chronic kidney disease. Clin Kidney J. (2023) 16:1751–65. doi: 10.1093/ckj/sfad076 PMC10616490 37915901

[16] Portales-Castillo I Simic PPTH. FGF-23, Klotho and Vitamin D as regulators of calcium and phosphorus: Genetics, epigenetics and beyond. Front Endocrinol (Lausanne). (2022) 13:992666. doi: 10.3389/fendo.2022.992666 36246903 PMC9558279

[17] Li S Yu L He A Liu Q. Klotho inhibits unilateral ureteral obstruction-induced endothelial-to-mesenchymal transition via TGF-β1/smad2/snail1 signaling in mice. Front Pharmacol. (2019) 10:348. doi: 10.3389/fphar.2019.00348 31024315 PMC6460315

[18] Gifford CC Lian F Tang J Costello A Goldschmeding R Samarakoon R. PAI-1 induction during kidney injury promotes fibrotic epithelial dysfunction via deregulation of klotho, p53, and TGF-β1-receptor signaling. FASEB J. (2021) 35:e21725. doi: 10.1096/fj.202002652RR 34110636 PMC8594377

[19] Ebert T Pawelzik S-C Witasp A Arefin S Hobson S Kublickiene K. Inflammation and premature ageing in chronic kidney disease. Toxins (Basel). (2020) 12(4):227. doi: 10.3390/toxins12040227 32260373 PMC7232447

[20] Karam S Rosner MH Sprangers B Stec R Malyszko J. Cancer therapy in patients with reduced kidney function. Nephrol Dial Transplant. (2024) 39:1976–84. doi: 10.1093/ndt/gfae142 38914465

[21] Jia M Han S Wang Y. Systemic immunoinflammatory indexes in albuminuric adults are negatively associated with α-klotho: evidence from NHANES 2007-2016. Ren Fail. (2024) 46:2385059. doi: 10.1080/0886022X.2024.2385059 39135529 PMC11328598

[22] Du R Liu J Tang X Chen Z Guan L Gao W. Correlation of neutrophil-to-lymphocyte ratio and platelet-to-lymphocyte ratio with serum α-klotho levels in US middle-aged and older individuals: Results from NHANES 2007-2016. Prev Med Rep. (2024) 46:102877. doi: 10.1016/j.pmedr.2024.102877 39309699 PMC11415581

[23] Peng X Hu Y Xu J Chen L Ren W Cai W. Inverse association between serum klotho levels and C-reactive protein levels in the US population: a cross-sectional study. BMC Cardiovasc Disord. (2024) 24:687. doi: 10.1186/s12872-024-04375-z 39614159 PMC11606063

[24] Qiu S Jiang Q Li Y. The association between pan-immune-inflammation value and chronic obstructive pulmonary disease: data from NHANES 1999-2018. Front Physiol. (2024) 15:1440264. doi: 10.3389/fphys.2024.1440264 39434724 PMC11491374

[25] Su Q Zhang W Li D Lan X Guo L Chen D. Association between blood lead levels and serum creatinine: a cross-sectional study. Int Urol Nephrol. (2024) 57(3):973–80. doi: 10.1007/s11255-024-04277-1 39522100

[26] Wu B Zhang C Lin S Zhang Y Ding S Song W. The relationship between the pan-immune-inflammation value and long-term prognoses in patients with hypertension: National Health and Nutrition Examination Study, 1999-2018. Front Cardiovasc Med. (2023) 10:1099427. doi: 10.3389/fcvm.2023.1099427 36937901 PMC10017977

[27] Chen P Tang Y Luo L Chen H He X. Lower serum Klotho level and higher systemic immune-inflammation index: an inverse correlation. BMC Geriatrics. (2023) 23:650. doi: 10.1186/s12877-023-04349-4 37821820 PMC10568854

[28] Levey AS Stevens LA Schmid CH Zhang YL Castro AF Feldman HI. A new equation to estimate glomerular filtration rate. Ann Intern Med. (2009) 150:604–12. doi: 10.7326/0003-4819-150-9-200905050-00006 PMC2763564 19414839

[29] Wen Z Liu X Zhang T. L-shaped association of systemic immune-inflammation index (SII) with serum soluble α-Klotho in the prospective cohort study from the NHANES database. Sci Rep. (2024) 14:13189. doi: 10.1038/s41598-024-64050-3 38851827 PMC11162490

[30] Centers for Disease Control and Prevention (CDC). Vital signs: current cigarette smoking among adults aged ≥18 years–United States, 2005-2010. MMWR Morb Mortal Wkly Rep. (2011) 60:1207–12. 21900875

[31] Kwak SG Kim JH. Central limit theorem: the cornerstone of modern statistics. Korean J Anesthesiol. (2017) 70:144–56. doi: 10.4097/kjae.2017.70.2.144 PMC5370305 28367284

[32] Duan M Zhao X Li S Miao G Bai L Zhang Q. Metabolic score for insulin resistance (METS-IR) predicts all-cause and cardiovascular mortality in the general population: evidence from NHANES 2001-2018. Cardiovasc Diabetol. (2024) 23:243. doi: 10.1186/s12933-024-02334-8 38987779 PMC11238348

[33] Xu B Wu Q La R Lu L Abdu FA Yin G. Is systemic inflammation a missing link between cardiometabolic index with mortality? Evidence from a large population-based study. Cardiovasc Diabetol. (2024) 23:212. doi: 10.1186/s12933-024-02251-w 38902748 PMC11191290

[34] Imai K Keele L Tingley D. A general approach to causal mediation analysis. Psychol Methods. (2010) 15:309–34. doi: 10.1037/a0020761 20954780

[35] Mardan M Zheng H Xu Q Song S Lu Z Deng H. Comprehensive analysis of Pan-Immune Inflammation and all-cause mortality in rheumatoid arthritis: a database-driven approach, 1999-2018. Front Immunol. (2025) 16:1549955. doi: 10.3389/fimmu.2025.1549955 39935476 PMC11811095

[36] Ruiz-Andres O Sanchez-Niño MD Moreno JA Ruiz-Ortega M Ramos AM Sanz AB. Downregulation of kidney protective factors by inflammation: role of transcription factors and epigenetic mechanisms. Am J Physiol Renal Physiol. (2016) 311:F1329–F40. doi: 10.1152/ajprenal.00487.2016 27760772

[37] Moreno JA Izquierdo MC Sanchez-Niño MD Suárez-Alvarez B Lopez-Larrea C Jakubowski A. The inflammatory cytokines TWEAK and TNFα reduce renal klotho expression through NFκB. J Am Soc Nephrol. (2011) 22:1315–25. doi: 10.1681/ASN.2010101073 PMC3137579 21719790

[38] Thurston RD Larmonier CB Majewski PM Ramalingam R Midura-Kiela M Laubitz D. Tumor necrosis factor and interferon-gamma down-regulate Klotho in mice with colitis. Gastroenterology. (2010) 138(4):1384–94. doi: 10.1053/j.gastro.2009.12.002 PMC3454518 20004202

[39] Martín-Núñez E Pérez-Castro A Tagua VG Hernández-Carballo C Ferri C Pérez-Delgado N. Klotho expression in peripheral blood circulating cells is associated with vascular and systemic inflammation in atherosclerotic vascular disease. Sci Rep. (2022) 12:8422. doi: 10.1038/s41598-022-12548-z 35590090 PMC9120199

[40] Panczyszyn-Trzewik P Misztak P Nowak G Sowa-Kucma M. Association of α-Klotho with regulation of Keap1/Nrf2/Interleukin-1 pathway and AMPA receptor trafficking in the brain of suicide victims. J Physiol Pharmacol. (2024) 75(3):10.26402. doi: 10.26402/jpp.2024.3.02 39042386

[41] Prud’homme GJ Wang Q. Anti-inflammatory role of the klotho protein and relevance to aging. Cells. (2024) 13(17):1413. doi: 10.3390/cells13171413 39272986 PMC11394293

[42] Salah TM Rabie MA El Sayed NS. Renoprotective effect of berberine in cisplatin-induced acute kidney injury: Role of Klotho and the AMPK/mtor/ULK1/Beclin-1 pathway. Food Chem Toxicol. (2025) 196:115179. doi: 10.1016/j.fct.2024.115179 39645019

[43] Abdelnaser M Attya ME El-Rehany MA Fathy M. Clemastine mitigates sepsis-induced acute kidney injury in rats; the role of α-Klotho/TLR-4/MYD-88/NF-κB/Caspase-3/p-P38 MAPK signaling pathways. Arch Biochem Biophys. (2025) 763:110229. doi: 10.1016/j.abb.2024.110229 39608427

[44] Bi F Chen F Li Y Wei A Cao W. Klotho preservation by Rhein promotes toll-like receptor 4 proteolysis and attenuates lipopolysaccharide-induced acute kidney injury. J Mol Med (Berl). (2018) 96:915–27. doi: 10.1007/s00109-018-1644-7 29730698

[45] Wang Y Xiong X Wang K Bao Y Zhang T Ainiwaer D. Peripheral Klotho protects the kidney and brain by regulating M2a/M2c macrophage polarization in d-gal-treated aged mice. Tissue Cell. (2023) 82:102049. doi: 10.1016/j.tice.2023.102049 36863110

[46] Wang Y Wang K Bao Y Zhang T Ainiwaer D Xiong X. The serum soluble Klotho alleviates cardiac aging and regulates M2a/M2c macrophage polarization via inhibiting TLR4/Myd88/NF-κB pathway. Tissue Cell. (2022) 76:101812. doi: 10.1016/j.tice.2022.101812 35597178

[47] Zhou W Chen M-M Liu H-L Si Z-L Wu W-H Jiang H. Dihydroartemisinin suppresses renal fibrosis in mice by inhibiting DNA-methyltransferase 1 and increasing Klotho. Acta Pharmacol Sin. (2022) 43:2609–23. doi: 10.1038/s41401-022-00898-3 PMC9525601 35347248

[48] Liu Y Bi X Xiong J Han W Xiao T Xu X. MicroRNA-34a promotes renal fibrosis by downregulation of klotho in tubular epithelial cells. Mol Ther. (2019) 27:1051–65. doi: 10.1016/j.ymthe.2019.02.009 PMC6520492 30853453

[49] Yu Y He J Li S Song L Guo X Yao W. Fibroblast growth factor 21 (FGF21) inhibits macrophage-mediated inflammation by activating Nrf2 and suppressing the NF-κB signaling pathway. Int Immunopharmacol. (2016) 38:144–52. doi: 10.1016/j.intimp.2016.05.026 27276443

[50] Xu B Cheng F Xue X. Klotho-mediated activation of the anti-oxidant Nrf2/ARE signal pathway affects cell apoptosis, senescence and mobility in hypoxic human trophoblasts: involvement of Klotho in the pathogenesis of preeclampsia. Cell Div. (2024) 19:13. doi: 10.1186/s13008-024-00120-2 38632651 PMC11025225

[51] Verde Z González-Moro JMR Chicharro LM Reinoso-Barbero L Bandrés F Gómez-Gallego F. A paradox: α-klotho levels and smoking intensity. Lung. (2017) 195:53–7. doi: 10.1007/s00408-016-9944-6 27752830

[52] Alvarez-Cienfuegos A Cantero-Nieto L Garcia-Gomez JA Robledo G González-Gay MA Ortego-Centeno N. FGF23-Klotho axis in patients with rheumatoid arthritis. Clin Exp Rheumatol. (2020) 38:50–7. 31025926

[53] Xiao Z King G Mancarella S Munkhsaikhan U Cao L Cai C. FGF23 expression is stimulated in transgenic α-Klotho longevity mouse model. JCI Insight. (2019) 4:(23):e132820. doi: 10.1172/jci.insight.132820 31801907 PMC6962016

[54] Long X Liu D Gao Q Ni J Qian L Ni Y. Bifidobacterium adolescentis alleviates liver steatosis and steatohepatitis by increasing fibroblast growth factor 21 sensitivity. Front Endocrinol (Lausanne). (2021) 12:773340. doi: 10.3389/fendo.2021.773340 35035378 PMC8756294

[55] Yu S Yang H Guo X Sun Y. Klotho attenuates angiotensin II−induced cardiotoxicity through suppression of necroptosis and oxidative stress. Mol Med Rep. (2021) 23:(1):66. doi: 10.3892/mmr.2020.11705 33215215 PMC7716407

[56] Wu C Lv C Chen F Ma X Shao Y Wang Q. The function of miR-199a-5p/Klotho regulating TLR4/NF-κB p65/NGAL pathways in rat mesangial cells cultured with high glucose and the mechanism. Mol Cell Endocrinol. (2015) 417:84–93. doi: 10.1016/j.mce.2015.09.024 26419931

[57] Prud’homme GJ Kurt M Wang Q. Pathobiology of the klotho antiaging protein and therapeutic considerations. Front Aging. (2022) 3:931331. doi: 10.3389/fragi.2022.931331 35903083 PMC9314780

[58] Xie B Cao K Li J Chen J Tang J Chen X. Hmgb1 inhibits Klotho expression and Malignant phenotype in melanoma cells by activating NF-κB. Oncotarget. (2016) 7:80765–82. doi: 10.18632/oncotarget.12623 PMC5348353 27779100

[59] Chung H Lee S Kim GA Kim WH. Down-expression of klotho in canine mammary gland tumors and its prognostic significance. PloS One. (2022) 17:e0265248. doi: 10.1371/journal.pone.0265248 35666743 PMC9170104

[60] Diakos CI Charles KA McMillan DC Clarke SJ. Cancer-related inflammation and treatment effectiveness. Lancet Oncol. (2014) 15:e493–503. doi: 10.1016/S1470-2045(14)70263-3 25281468

[61] Lattanzi R Severini C Miele R. Prokineticin 2 in cancer-related inflammation. Cancer Lett. (2022) 546:215838. doi: 10.1016/j.canlet.2022.215838 35921971

[62] Kao T-W Huang C-C Leu H-B Yin W-H Tseng W-K Wu Y-W. Inflammation and renal function decline in chronic coronary syndrome: a prospective multicenter cohort study. BMC Cardiovasc Disord. (2023) 23:564. doi: 10.1186/s12872-023-03565-5 37974082 PMC10655285

[63] Wang Z Xue H Sun Y Wang Q Sun W Zhang H. Deciphering the biological aging impact on alveolar bone loss: insights from α-klotho and renal function dynamics. J Gerontol A Biol Sci Med Sci. (2024) 79:(9):glae172. doi: 10.1093/gerona/glae172 38995226

[64] Guo A Cao J Wu C Ding S. Uric acid mediates the association between testosterone and α-Klotho among males: results from the NHANES 2013-2016. Int Urol Nephrol. (2024) 57(3):939–46. doi: 10.1007/s11255-024-04262-8 39487906

[65] Gherghina M-E Peride I Tiglis M Neagu TP Niculae A Checherita IA. Uric acid and oxidative stress-relationship with cardiovascular, metabolic, and renal impairment. Int J Mol Sci. (2022) 23(6):3188. doi: 10.3390/ijms23063188 35328614 PMC8949471

[66] Słomiński B Ryba-Stanisławowska M Skrzypkowska M Gabig-Cimińska M Myśliwiec M. A new potential mode of cardiorenal protection of KLOTHO gene variability in type 1 diabetic adolescents. J Mol Med (Berl). (2020) 98:955–62. doi: 10.1007/s00109-020-01918-7 PMC7343757 32435919

[67] Lu H Yao H Zou R Chen X Xu H. Galangin suppresses renal inflammation via the inhibition of NF-κB, PI3K/AKT and NLRP3 in uric acid treated NRK-52E tubular epithelial cells. BioMed Res Int. (2019) 2019:3018357. doi: 10.1155/2019/3018357 31240210 PMC6556363

[68] Jiang X Wu Q Opoku YK Zou Y Wang D Hu C. Fibroblast growth factor 21 attenuates the progression of hyperuricemic nephropathy through inhibiting inflammation, fibrosis and oxidative stress. Basic Clin Pharmacol Toxicol. (2022) 131:474–86. doi: 10.1111/bcpt.v131.6 36126111

[69] Wang Q Su W Shen Z Wang R. Correlation between soluble α-klotho and renal function in patients with chronic kidney disease: A review and meta-analysis. BioMed Res Int. (2018) 2018:9481475. doi: 10.1155/2018/9481475 30159331 PMC6109492

[70] Donate-Correa J Ferri CM Martín-Núñez E Pérez-Delgado N González-Luis A Mora-Fernández C. Klotho as a biomarker of subclinical atherosclerosis in patients with moderate to severe chronic kidney disease. Sci Rep. (2021) 11:15877. doi: 10.1038/s41598-021-95488-4 34354161 PMC8342510

[71] Zhou X Ji S Chen L Liu X Deng Y You Y. Gut microbiota dysbiosis in hyperuricaemia promotes renal injury through the activation of NLRP3 inflammasome. Microbiome. (2024) 12:109. doi: 10.1186/s40168-024-01826-9 38907332 PMC11191305

[72] Wu M Ma Y Chen X Liang N Qu S Chen H. Hyperuricemia causes kidney damage by promoting autophagy and NLRP3-mediated inflammation in rats with urate oxidase deficiency. Dis Model Mech. (2021) 14(3):dmm048041. doi: 10.1242/dmm.048041 33648977 PMC8015218

