# Supplementary material for: Association between pan-immune-inflammation value and Serum Klotho levels: a cross-sectional analysis of renal function mediation
Source: Front Immunol. 2025 May 30;16:1567367. doi: 10.3389/fimmu.2025.1567367 (PMC12163038; doi:10.3389/fimmu.2025.1567367)
Supplement: Supplementary file 1 [file DataSheet1.pdf]

Supplementary Table 1. Variance Inflation Factors of Variables.

	VIF
PIV	1
Age	1.2
Gender	1.1
Race	1.1
Education Level	1.3
Marital Status	1.1
PIR	1.4
BMI	1.1
Smoking Status	1.2
Alcohol Consumption	1.2
Hypertension	1.2
Diabetes	1.1
High Cholesterol	1.1
Cancer	1.1

Supplementary Table 2. Mediation Analysis for the Association Between PIV and Klotho Levels.

**Mediator**	**β (95% CI) *P*-value**			**% (95% CI) *P*-value**
	**Total effect**	**Mediation effect**	**Direct effect**	**Proportion mediated**
Cr	-22.43 (-28.65, -15.69) <0.0001	-1.23 (-1.89, -0.62) <0.0001	-21.20 (-27.27, -14.49) <0.0001	5.49 (2.62, 9.31) 0.0001
BUN	-22.43 (-28.65, -15.69) <0.0001	-1.49 (-2.29, -0.77) <0.0001	-20.94 (-26.91, -14.32) <0.0001	6.64 (3.33, 11.06) 0.0001
UA	-22.43 (-28.65, -15.69) <0.0001	-1.87 (-2.79, -1.04) <0.0001	-20.56 (-26.35, -14.03) <0.0001	8.32 (4.55, 13.30) <0.0001
eGFR	-22.43 (-28.65, -15.69) <0.0001	-1.55 (-2.33, -0.79) <0.0001	-20.88 (-26.88, -14.41) <0.0001	6.91 (3.43, 11.68) <0.0001

Adjusted for potential confounders as specified in Model 3.

Supplementary Table 3. Baseline Characteristics of Study Participants Stratified by Cancer Status.

**Variables**	**Cancer Survivors**	**Non-Cancer Participants**	***P*-value**
**Klotho (pg/mL)**	813.74±8.55	849.64±5.08	<0.0001
**PIV**	320.07±6.58	299.07±3.44	0.0059
**Age (years)**	62.69±0.38	55.13±0.15	<0.0001
**Gender (%)**			0.1209
Male	45.57	48.08	
Female	54.43	51.92	

**Race (%)**			<0.0001
Mexican American	2.4	7.41	
Other Hispanic	2.16	5.12	
Non-Hispanic White	86.57	70.84	
Non-Hispanic Black	4.57	9.69	
Other Race	4.31	6.93	
**Education Level (%)**			<0.0001
Less than High School	3.72	6.55	
High School or GED	26.13	33.46	
More than High School	70.15	60	
**Marital Status (%)**			0.0046
Married/Living with Partner	69.82	70.52	
Widowed/Divorced/Separated	22.7	19.23	
Never married	7.47	10.25	
**PIR**	3.42±0.07	3.23±0.05	0.0006
**BMI (kg/m²)**	29.46±0.21	29.54±0.10	0.7214
**Smoking Status (%)**			<0.0001
Current	18.72	16.69	
Former	28.35	38.88	
Never	52.93	44.43	
**Alcohol Consumption (%)**			0.5678
Yes	78.22	77.36	
No	21.78	22.64	
**Hypertension (%)**			<0.0001
Yes	52.51	39.67	
No	47.49	60.33	
**Diabetes (%)**			0.003
Yes	16.12	13	
No	80.39	84.46	
Borderline	3.49	2.54	
**High Cholesterol (%)**			<0.0001
Yes	57.32	46.08	
No	42.68	53.92	
**Cr (mg/dL)**	0.93±0.01	0.90±0.00	0.0047
**BUN (mg/dL)**	15.34±0.22	14.01±0.09	<0.0001
**UA (mg/dL)**	5.48±0.05	5.47±0.02	0.8592
**eGFR (mL/min/1.73m²)**	80.26±0.70	87.83±0.31	<0.0001

Abbreviations: PIV: pan-immune-inflammation value; BMI: body mass index; PIR: Family Income to Poverty Ratio; Cr: serum creatinine; BUN: blood urea nitrogen; UA: Serum uric acid; eGFR: estimated

glomerular filtration rate.

Supplementary Table 4. Association between PIV Quartiles and Klotho levels Stratified by Cancer Status.

**PIV**	**β (95% CI) *P*-value**		
	Model 1	Model 2	Model 3
**Cancer Survivors**			
Q1	**Reference**	**Reference**	**Reference**
Q2	-99.15 (-154.71, -43.60)<br>0.0008	-98.49 (-154.65, -42.32)<br>0.0010	-13.96 (-78.56, 50.65)<br>0.6735
Q3	-100.25 (-154.29, -46.20)<br>0.0005	-90.83 (-145.44, -36.21)<br>0.0017	-51.98 (-114.30, 10.34)<br>0.1076
Q4	-109.21 (-171.77, -46.65)<br>0.0010	-98.00 (-158.28, -37.72)<br>0.0022	-59.13 (-116.43, -1.83)<br>0.0478
P for trend	0.0034	0.0100	0.0233
**Non-Cancer Participants**			
Q1	**Reference**	**Reference**	**Reference**
Q2	-34.77 (-56.97, -12.56)<br>0.0030	-29.50 (-52.48, -6.52)<br>0.0141	-39.89 (-66.04, -13.74)<br>0.0041
Q3	-48.45 (-71.79, -25.10)<br>0.0001	-42.96 (-66.93, -18.99)<br>0.0008	-45.57 (-71.23, -19.90)<br>0.0010
Q4	-75.71 (-97.54, -53.88)<br><0.0001	-66.16 (-88.70, -43.62)<br><0.0001	-67.82 (-94.34, -41.29)<br><0.0001
P for trend	<0.0001	<0.0001	<0.0001

Model 1: Unadjusted.

Model 2: Adjusted for age, sex, race.

Model 3: Adjusted for age, sex, race, PIR, BMI, education level, marital status, smoking status, drinking status, hypertension, diabetes, and high cholesterol.

Abbreviations: PIV: pan-immune-inflammation value; PIR: Family Income to Poverty Ratio; BMI: body mass index.
